# Accurate H-atom parameters from X-ray diffraction data

**DOI:** 10.1107/S2052252514018612

**Published:** 2014-08-29

**Authors:** Louis J. Farrugia

**Affiliations:** a School of Chemistry, University of Glasgow, Glasgow G12 8QQ, Scotland

**Keywords:** aspherical atom-partitioning, Hirshfeld atom refinement, density functional theory

## Abstract

The difficulties in defining the positions and thermal parameters for hydrogen atoms using X-ray diffraction data alone are discussed.

The difficulties in defining the positions and thermal parameters for hydrogen atoms using X-ray diffraction data alone are well known and arise from the lack of any core electron density. In this commentary, I will be focusing on single-crystal diffraction experiments, though similar considerations also apply to powder diffraction experiments. The X-ray scattering from the H-atom valence electron density falls off significantly with resolution and one ‘trick’ in use for a considerable time is to use a truncated data set to obtain a less-biased set of H-atom positions. Even in the most favourable cases, however, there remain intrinsic and well understood errors in the derived positions, which manifest themselves in shorter *X*—H distances than those determined by neutron diffraction. This latter diffraction technique does not suffer from the same problems as X-ray diffraction regarding the derived H-atom parameters, but has itself serious limitations, not least the expensive nature of neutron generation and the requirement of relatively large single crystals. For all these reasons, the ‘normal’ refinement programs for X-ray data which use spherical atomic scattering factors, such as *SHELXL* (Sheldrick, 2008 ▶), all allow for the fixing of H-atom positions and thermal parameters at standardized values.

For most X-ray diffraction studies (in which the atomic arrangement is the most important information needed), such compromises are satisfactory, but for highly accurate work, such as in experimental charge density studies, it is necessary to make adjustments for these deficiencies. Non-spherical scattering factors for atoms are essential (Stewart, 1969 ▶) and one common method is to approximate these using a multipole expansion, such as the well known Hansen–Coppens model (Hansen & Coppens, 1978 ▶). Nevertheless, the above mentioned problems remain for the H atom, and it is not generally possible to expand the H-atom asphericity above the dipole level. The multipole model requires many more refinable parameters, but usually results in refined *X*—H distances which are much closer to the neutron-determined ‘true’ distances. Normally, however, it remains common practice to fix the *X*—H distances to averaged neutron-determined distances. A number of studies have shown that an anisotropic description of the H-atom thermal motion is essential to obtain an accurate representation of the static electron density (Spackman *et al.*, 2007 ▶). H-atom anisotropic displacement parameters (a.d.p.’s) cannot be obtained directly from the X-ray data, but may be estimated by a number of methods (Madsen, 2012 ▶). The use of such estimated a.d.p.’s as fixed contributions is becoming the norm in experimental charge density studies. For these methods to work effectively, it is necessary to obtain X-ray diffraction data to high resolution [generally sin(θ)/λ > 1.0 Å^−1^], which itself introduces significant extra restrictions to the applicability of the technique.

Jayatilaka and coworkers (Capelli *et al.*, 2014 ▶) have recently shown that another approach is possible, whereby accurately refined H-atom positions and a.d.p.’s can be obtained from low resolution X-ray diffraction data [sin(θ)/λ ≃ 0.6 Å^−1^] obtained in standard data collections. Their method relies on using individual aspherical scattering factors computed by Fourier transformation of the aspherical Hirshfeld atomic densities, themselves obtained from a DFT calculation on a pseudo-crystal. The method is currently limited to molecular crystals, as a molecular wavefunction is computed, but by using fully periodic wavefunctions it should be possible to extend the method to network solids. This approach has been called ‘Hirshfeld Atom Refinement’, since the relatively simple Hirshfeld stockholder partitioning scheme (Hirshfeld, 1977 ▶) is used to compute the aspherical atomic electron densities from the molecular density. A new development described in the paper by Jayatilaka and coworkers (Capelli *et al.*, 2014 ▶) is to use an iterative approach. The initially determined atomic coordinates are used to compute a pseudo-crystal wavefunction from which the Hirshfeld atomic electron densities are determined. A Fourier transform affords the aspherical atomic scattering factors, which are then used in a conventional crystallographic least-squares refinement to obtain modified atomic coordinates, which are then fed back into a new wavefunction calculation. The process continues until convergence. Given good quality X-ray diffraction data, it is possible to freely refine all the H-atom positions and anisotropic thermal parameters. Jayatilaka and coworkers (Capelli *et al.*, 2014 ▶) have shown that the H-atom parameters and their errors, obtained by this Hirshfeld Atom Refinement, have a precision and accuracy which compares very favourably with neutron diffraction experiments.

This is a very exciting result. Although there are inevitably some limitations in the current implementation (the method is quite computer-intensive), the ability to obtain accurate H-atom parameters from low resolution X-ray diffraction data alone is a great step forward. Jayatilaka and coworkers (Capelli *et al.*, 2014 ▶) compare the neutron and Hirshfeld Atom Refinements on a dipeptide Gly–l-Ala for which excellent synchrotron X-ray data were available and also discuss the method using previously published data on ammonia, urea and benzene. It remains to be seen how factors such as less than optimal quality diffraction data affect the results of a Hirshfeld Atom Refinement.
